# Household Economic Consequences of Rheumatic Heart Disease in Uganda

**DOI:** 10.3389/fcvm.2021.636280

**Published:** 2021-07-30

**Authors:** Chinonso C. Opara, Yuxian Du, Yoshito Kawakatsu, Jenifer Atala, Andrea Z. Beaton, Rosemary Kansiime, Miriam Nakitto, Emma Ndagire, Haddy Nalubwama, Emmy Okello, David A. Watkins, Yanfang Su

**Affiliations:** ^1^Department of Medicine, University of Washington, Seattle, WA, United States; ^2^Hutchinson Institute for Cancer Outcome Research, Fred Hutchinson Cancer Research Center, Seattle, WA, United States; ^3^Department of Global Health, University of Washington, Seattle, WA, United States; ^4^Department of Community-Based Rehabilitation Sciences, Graduate School of Biomedical Sciences, Nagasaki University, Nagasaki, Japan; ^5^Department of RHD Research, Uganda Heart Institute, Kampala, Uganda; ^6^Department of Cardiology, The Heart Institute, Cincinnati Children's Hospital Medical Center, Cincinnati, OH, United States; ^7^Department of Pediatrics, School of Medicine, University of Cincinnati, Cincinnati, OH, United States; ^8^Division of Cardiology, Uganda Heart Institute, Kampala, Uganda

**Keywords:** rheumatic heart disease, catastrophic health expenditure, universal health coverage, cost of illness, household survey

## Abstract

**Background:** Rheumatic heart disease (RHD) has declined dramatically in wealthier countries in the past three decades, but it remains endemic in many lower-resourced regions and can have significant costs to households. The objective of this study was to quantify the economic burden of RHD among Ugandans affected by RHD.

**Methods:** This was a cross-sectional cost-of-illness study that randomly sampled 87 participants and their households from the Uganda National RHD registry between December 2018 and February 2020. Using a standardized survey instrument, we asked participants and household members about outpatient and inpatient RHD costs and financial coping mechanisms incurred over the past 12 months. We used descriptive statistics to analyze levels and distributions of costs and the frequency of coping strategies. Multivariate Poisson regression models were used to assess relationships between socioeconomic characteristics and utilization of financial coping mechanisms.

**Results:** Most participants were young or women, demonstrating a wide variation in socioeconomic status. Outpatient and inpatient costs were primarily driven by transportation, medications, and laboratory tests, with overall RHD direct and indirect costs of $78 per person-year. Between 20 and 35 percent of households experienced catastrophic healthcare expenditure, with participants in the Northern and Western Regions 5–10 times more likely to experience such hardship and utilize financial coping mechanisms than counterparts in the Central Region, a wealthier area. Increases in total RHD costs were positively correlated with increasing use of coping behaviors.

**Conclusion:** Ugandan households affected by RHD, particularly in lower-income areas, incur out-of-pocket costs that are very high relative to income, exacerbating the poverty trap. Universal health coverage policy reforms in Uganda should include mechanisms to reduce or eliminate out-of-pocket expenditures for RHD and other chronic diseases.

## Introduction

Rheumatic heart disease (RHD) has declined dramatically in wealthy countries over the past three decades, in part due to penicillin-based preventive measures and improvements in social determinants of infectious diseases (1). However, RHD continues to be endemic in lower-resourced regions such as Southeast Asia and Sub-Saharan Africa (2). Rheumatic heart disease is thought to pose a severe threat to the economic well-being of affected households because of its longitudinal nature and associated recurrent out-of-pocket (OOP) costs, including OOP costs of advanced medical and surgical care. The magnitude of these costs is particularly high in low-income countries like Uganda, where health services that are not financed by development assistance partnerships are often paid for OOP (3).

High OOP costs and their consequences, including catastrophic health expenditure, borrowing, and selling assets to pay for healthcare, and intra-household labor substitution, threaten to keep households in the so-called “poverty trap” (4). More broadly speaking, high rates of medical impoverishment threaten the macroeconomic growth agenda in low-income countries. Indeed, reducing OOP payments has become a key feature of universal health coverage (UHC) reforms. Unfortunately, the scarcity of public resources constrains the ability of many governments to provide adequate financial protection for all but the highest-priority health conditions and services—and to date, RHD has typically been neglected in national priorities (5).

Even though there are good theoretical reasons as to why RHD could be associated with excess household economic burden, there are only a handful of primary costing studies to date that have attempted to quantify the economic burden of RHD from the societal, health sector, and household perspectives (6–12). Only one study (non-peer reviewed) has assessed the household economic impact of RHD in a country where the condition remains endemic (South Africa) (13), and the health system and financing capabilities of South Africa are relatively advanced as compared to most other African nations, limiting the applicability of this study's findings to different settings.

In 2018, the 71^st^ World Health Assembly adopted a global resolution on RHD (14), making this condition a high priority for national UHC systems for the first time. In response to this resolution, the Uganda Ministry of Health has begun to work with the Uganda Heart Institute to develop a national RHD strategy and policies. Around the same time, the American Heart Association made a 4-year investment in RHD research in Uganda, focusing on filling gaps in epidemiological and health services research. The present study, conducted under the auspices of the above collaborations, sought to quantify the economic burden of RHD in Uganda from the household perspective. On a local level, this study aimed to guide the allocation of resources in Uganda to address RHD, particularly by understanding the potential scope for public finance of RHD-related healthcare.

## Methods

### Overview

This study used the cost-of-illness method (15). We used a prevalence-based approach to cost estimation and thus designed the study as a cross-sectional survey. In brief, we collected data on direct and indirect RHD-related costs among 87 Ugandan households affected by RHD, looking at all costs incurred over the previous 12 months. We also estimated the prevalence of catastrophic health expenditure among these households and inquired about the use of specific coping mechanisms (e.g., selling assets) to smooth consumption over time.

### Recruitment of Subjects

Participants in this study were recruited from the Uganda National RHD Registry. This registry was established in 2010 as a central database for all patients diagnosed with RHD clinically and by echocardiogram in Uganda (16). As of this writing, the registry has enrolled 2,727 participants. For our study, we sampled from among registry enrollees who were receiving RHD care at one of three referral hospitals that served the district in which they lived. The hospitals chosen were Lira Regional Referral Hospital (Lira district, Northern Region), Mbarara Regional Referral Hospital (Mbarara district, Western Region), and Mulago National Referral Hospital (Wakiso district, Central Region).

We pre-screened the Uganda National RHD Registry database for enrollees who were receiving care at the three hospitals mentioned previously and did a stratified random sample of subjects for inclusion in this study. Edited to reflect that we have a few subjects under age 10. Minors were required to provide assent and informed consent from their guardians. There were no clinical exclusion criteria.

Our original target sample size was 100 subjects, distributed approximately equally across the three sites (sampling strata). This target had been chosen to obtain a precision of ±5% on critical descriptive statistics. Eighty-seven subjects had been recruited as of March 2020, at which time the ethical review committees of the responsible institutions suspended nearly all research activities due to the SARS-CoV-2 outbreaks in the United States and Uganda. Because of the uncertain timeframe for resumption of study activities, we decided to close out the study and analyze data for the 87 subjects who were already enrolled.

### Data Collection Procedures

Eligible subjects were contacted by a research nurse who explained the reason they had been identified as a potential participant and described the study objective and procedures. Subjects agreeing to participate were scheduled to undergo an in-person survey that was usually conducted at the subject's place of residence. Some subjects (e.g., minors) did not have sufficient information on household finances; in these cases, a household representative with knowledge of finances was also asked to participate in the survey. Written informed consent (and assent, when relevant) was obtained from all individuals who provided responses to the surveys.

After documentation of consent, a research nurse administered a standardized survey instrument that contained two modules, an “individual” module focused on the costs incurred by the patient receiving care for RHD and a “household” module focused on household demographics, income, expenditures, and assets. The survey instrument was adapted from previous studies and was piloted on several subjects prior to finalization. Supplementary Material contains the entire survey instrument.

Surveys were conducted from December 2018 through February 2020 and in the participants' preferred language (Luo, Runyankore, Luganda, or English). Following survey completion, participating households were reimbursed 20,000 Ugandan Shillings (about 5.5 United States dollars) for their time.

### Data Analysis

Cost estimates obtained in the surveys were initially recorded in current Ugandan Shillings. The research team then standardized these costs to 2019 mid-year United States dollars (US$) using exchange rates and consumer price indices from the most recent World Development Indicators dataset (2020 update) (17).

We first analyzed the survey data using descriptive statistics, linking every inpatient or outpatient episode for RHD that was recalled over the previous 12 months back to a unique patient/household identifier. Costs of healthcare episodes were divided into direct medical costs, direct non-medical costs, and indirect costs (4). We further disaggregated direct medical costs into those due to laboratory tests, consultations, medicines, and in the case of inpatient care, bed tariffs. We disaggregated direct non-medical costs into those due to transportation, accommodation, and food expenses. Indirect (i.e., opportunity) costs were calculated using reported time spent receiving or providing RHD-related care and were disaggregated into costs of ill participants and their caretakers, respectively. The human capital approach was used to estimate indirect costs: reported hours of work missed were multiplied by the Uganda national minimum wage (converted into an hourly rate) (18, 19).

We also estimated the prevalence of catastrophic health expenditure (CHE) among study subjects. We computed total annual direct expenditure on RHD-related care for each subject and compared this expenditure to total annual household expenditure. Two thresholds for CHE were used: RHD expenditure greater than or equal to 10% or 25% of household expenditure (20).

Next, we estimated the prevalence of common coping mechanisms that occurred following utilization of RHD-related care. We asked participants about the use of three mechanisms: (i) taking out one or more loans, (ii) receiving financial assistance from family or friends, and (iii) selling assets. Again, the recall window for these events was the previous 12 months.

Finally, we assessed the relationship between households' use of coping mechanisms and the demographic and socioeconomic characteristics of household members affected by RHD. We used multivariate Poisson regression models to test a pre-specified set of covariates (see **Table 4** for list). The final adjusted model included all covariates.

### Other Information

Survey data were managed by a REDCap account hosted by Children's National Medical Center and were exported to Microsoft Excel (v2104) and R (v3.6.3) for data cleaning and analysis (21).

The sponsor of this research was not involved in the design, review, collection of data, analysis, and interpretation of data, or drafting of this manuscript.

## Results

### Characteristics of Study Subjects and Households

We conducted surveys of 87 individuals with RHD and their households. Of these, 33 were residing in the Northern Region, 20 in the Western Region, and 34 in the Central Region. Table 1 summarizes the demographic characteristics of the patients and households. The “typical” participant was a young adult woman who achieved at least primary or secondary level education but who was currently unemployed and did not have private health insurance. Notably, socioeconomic characteristics varied widely across participants from the three regions. Participants living in the Northern Region generally demonstrated the lowest income and educational attainment, and participants living in the Central Region the highest.

**Table 1:** Baseline characteristics of sampled participants.

	**Northern region**	**Western region**	**Central region**	**All regions**
	**(*n* = 33)**	**(*n* = 20)**	**(*n* = 34)**	**(*n* = 87)**
**AGE**				
Mean (*S.D*.)^a^	22 (17)	28 (16)	34 (13)	28 (16)
Female, %	64	60	79	69
**EDUCATION**, ***n*****(%)**				
None	5 (15)	1 (5)	3 (9)	9 (10)
Primary only	23 (70)	8 (40)	7 (21)	38 (44)
Secondary or more	5 (15)	11 (55)	24 (71)	40 (46)
**EMPLOYMENT**, ***n*****(%)**				
Unemployed	28 (85)	13 (65)	24 (71)	65 (75)
Self-employed	5 (15)	4 (20)	4 (12)	13 (15)
Formally employed (full-time/part-time)	0 (0)	3(15)	6 (18)	9 (10)
**HOUSEHOLD ASSETS**, ***n*****(%)**				
Electricity	5 (15)	6 (30)	26 (77)	37 (43)
Mobile phone	30 (91)	19 (95)	32 (94)	81 (93)
Any vehicle	8 (24)	10 (50)	14 (41)	32 (37)
**HOUSEHOLD SIZE**				
Mean (*S.D*.)	7.0 (4.0)	5.2 (1.7)	1.2 (1.2)	4.3 (3.7)
**AVERAGE 30-DAY HOUSEHOLD EXPENDITURES (USD)^b^, MEAN (*****S.D***.**)**				
Non-food expenditure	44.80 (74.49)	45.39 (59.09)	94.18 (106.43)	64.68 (88.64)
Food	37.53 (58.65)	56.77 (46.06)	81.22 (56.75)	58.77 (57.91)
**MONTHLY HOUSEHOLD INCOME PER PERSON (USD)^b^**				
Mean (*S.D*.)	27.81 (57.64)	40.39 (49.18)	276.81 (506.04)	126.28 (335.40)
**PRIVATE HEALTH INSURANCE**				
*n* (%)	0 (0)	0 (0)	4 (12)	4 (5)

a *Standard deviation*,

b *currency reported in 2019 USD*.

Among households included in this study, the average monthly income per person was US$ 130, though it varied from US$ 28 in the Northern Region to US$ 280 in the Central Region. The average monthly household expenditure was US$ 123 in total, of which 52% were non-food expenditures. Less than half of households had electricity, 37% owned a vehicle (car, motorcycle), and 93% had at least one mobile phone.

### Direct and Indirect Costs Incurred

Direct and indirect costs were incurred across 27 inpatient visits from 21 participants and 408 outpatient visits among all the 87 participants. Overall annual costs were estimated at US$ 78 per person per year, inclusive of both direct and indirect costs.

**Figure 1:**
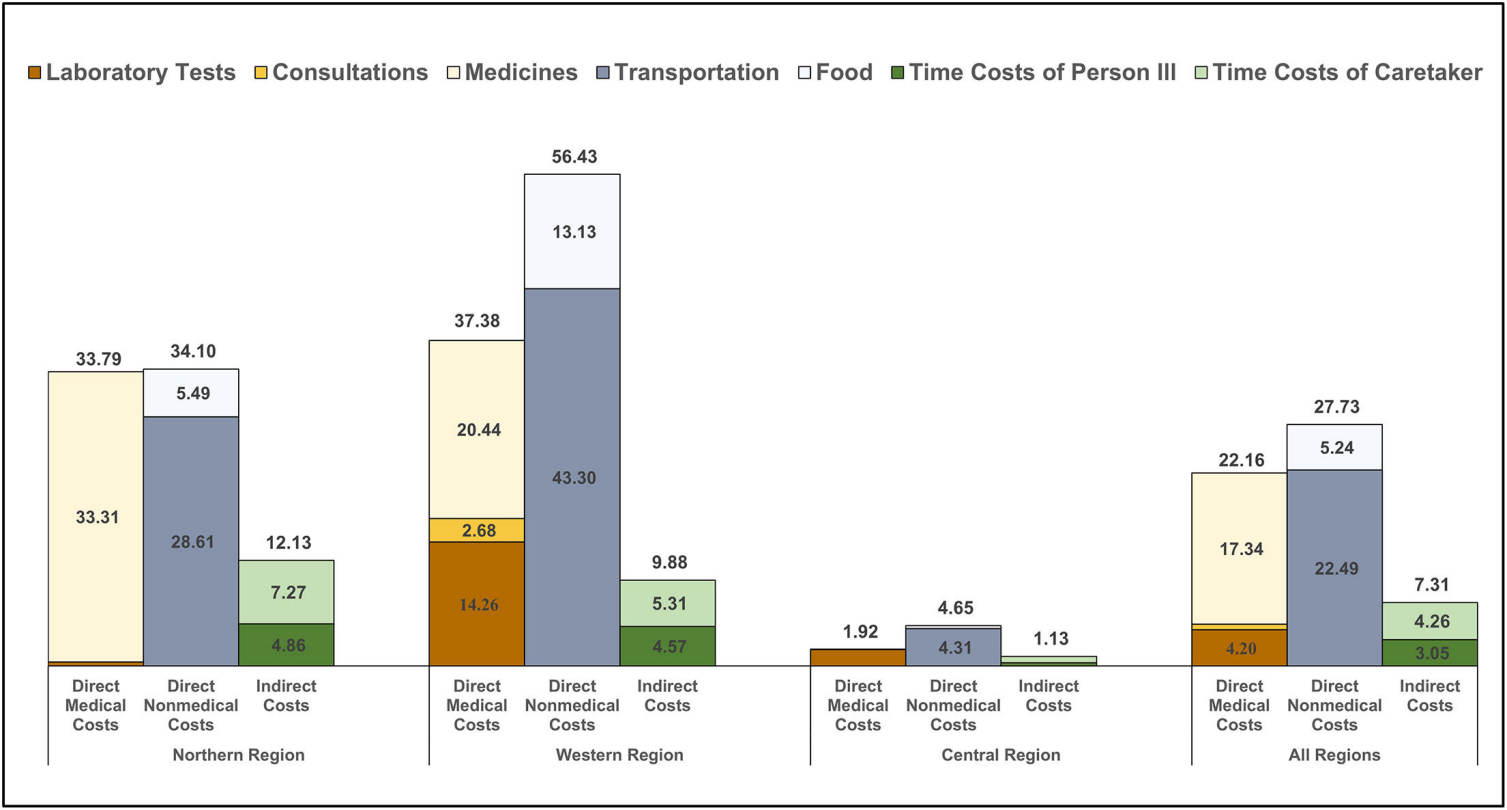
Outpatient costs incurred from receiving care for RHD. The bar graphs show the level and distribution of average costs per person per year for outpatient encounters (*n* = 408). Thirty-three participants from the Northern Region represented 214 visits; 20 participants from the Western Region represented 147 visits; and 34 participants from the Central Region represented 47 visits. Costs are disaggregated by region and by component and include direct and indirect costs, though the latter were low for outpatient care. Costs are presented in 2019 United States dollars.

As highlighted in Table 2 and Figure 1, overall direct costs of outpatient care were comprised predominately of transportation and medications expenses and, to a lesser extent, food and laboratory tests expenses. Notably, OOP medication costs were lower in the Central Region, which probably relates to the greater availability of free medications at public facilities in the Region.

**Table 2:** Costs^a^ accrued from seeking RHD care.

	**Outpatient**		**Inpatient**		**Combined**	
	**Mean**	**Percentage of subtotal (%)**	**Mean**	**Percentage of subtotal (%)**	**Mean**	**Percentage of subtotal (%)**
**ALL REGIONS (** ***N*** **=** **87)**						
**Direct Medical Costs**						
Laboratory tests	4.20	19	3.29	32	7.49	23
Consultations	0.62	3	1.15	11	1.77	5
Medicines	17.34	78	4.37	42	21.71	67
Bed tariffs	N/A	N/A	1.48	14	1.48	5
*Subtotal*	*22.16*	*39*	*10.28*	*50*	*32.45*	*42*
**Direct Non-Medical Costs**						
Transportation	22.49	81	3.17	47	25.66	74
Accommodation	–	0	1.41	21	1.41	4
Food and other expenses	5.24	19	2.15	32	7.39	21
*Subtotal*	*27.73*	*48*	*6.74*	*33*	*34.46*	*44*
**Indirect Costs**						
Time costs of person ill	3.05	42	2.84	82	5.89	55
Time cost of caretakers	4.26	58	0.64	18	4.90	45
*Subtotal*	*7.31*	*13*	*3.48*	*17*	*10.79*	*14*
**TOTAL**	57.20	74	20.50	26	77.70	100
**NORTHERN REGION (N** **=** **33)**						
**Direct Medical Costs**						
Laboratory tests	0.49	1	4.53	30	5.01	10
Consultations	–	0	0.12	1	0.12	0
Medicines	33.31	99	9.98	67	43.28	89
Bed tariffs	N/A	N/A	0.36	2	0.36	1
*Subtotal*	*33.79*	*42*	*14.99*	*50*	*48.78*	*44*
**Direct Non-Medical costs**						
Transportation	28.61	84	4.27	52	33	78
Accommodation	–	0	–	0	–	0
Food and other expenses	5.49	16	3.97	48	9	22
*Subtotal*	*34.10*	*43*	*8.24*	*28*	*42.34*	*39*
**Indirect Costs**						
Time costs of person ill	4.86	40	5.32	81	10.18	55
Time cost of caretakers	7.27	60	1.21	19	8.49	45
*Subtotal*	*12.13*	*15*	*6.54*	*22*	*18.67*	*17*
**TOTAL**	80.03	73	29.76	27	109.79	100
**WESTERN REGION (** ***n*** **=** **20)**						
**Direct Medical Costs**						
Laboratory tests	14.26	38	–	0	14.26	37
Consultations	2.68	7	0.27	18	2.94	8
Medicines	20.44	55	1.20	82	21.65	56
Bed tariffs	N/A	N/A	–	0	–	0
*Subtotal*	*37.38*	*36*	*1.47*	*22*	*38.85*	*35*
**Direct Non-Medical costs**						
Transportation	43.30	77	5.08	100	48.39	79
Accommodation	–	0	–	0	–	0
Food and other expenses	13.13	23	–	0	13.13	21
*Subtotal*	*56.43*	*54*	*5.08*	*76*	*61.52*	*56*
**Indirect Costs**						
Time costs of person ill	4.57	46	0.17	100	4.75	47
Time cost of caretakers	5.31	54	–	0	5.31	53
*Subtotal*	*9.88*	*10*	*0.17*	*3*	*10.05*	*9*
**TOTAL**	103.69	94	6.73	6	110.42	100
**CENTRAL REGION (** ***n*** **=** **34)**						
**Direct Medical Costs**						
Laboratory tests	1.89	98	4.01	37	5.90	46
Consultations	–	0	2.68	25	2.68	21
Medicines	0.03	2	0.79	7	0.82	6
Bed tariffs	N/A	N/A	3.42	31	3.42	27
*Subtotal*	*1.92*	*25*	*10.90*	*56*	*12.82*	*47*
**Direct Non-Medical Costs**						
Transportation	4.31	93	0.98	16	5.29	48
Accommodation	–	0	3.62	58	3.62	33
Food and other expenses	0.35	7	1.65	26	2.00	18
*Subtotal*	*4.65*	*60*	*6.26*	*32*	*10.91*	*40*
**Indirect Costs**						
Time costs of person ill	0.41	36	2.00	81	2.40	67
Time cost of caretakers	0.72	64	0.46	19	1.18	33
*Subtotal*	*1.13*	*15*	*2.46*	*13*	*3.58*	*13*
**TOTAL**	7.70	28	19.61	72	27.31	100

a *Costs reported in 2019 USD as per person per year. Itemized costs add up to 100% of their respective sub-divisions. For example, laboratory tests, consultations, medicines and bed tariffs represent 100% of direct medical costs. Combining subtotals of direct medical, direct non-medical and indirect costs adds up to 100% of total costs. Likewise, combining outpatient and inpatient costs adds up to 100% of total costs*.

**Figure 2:**
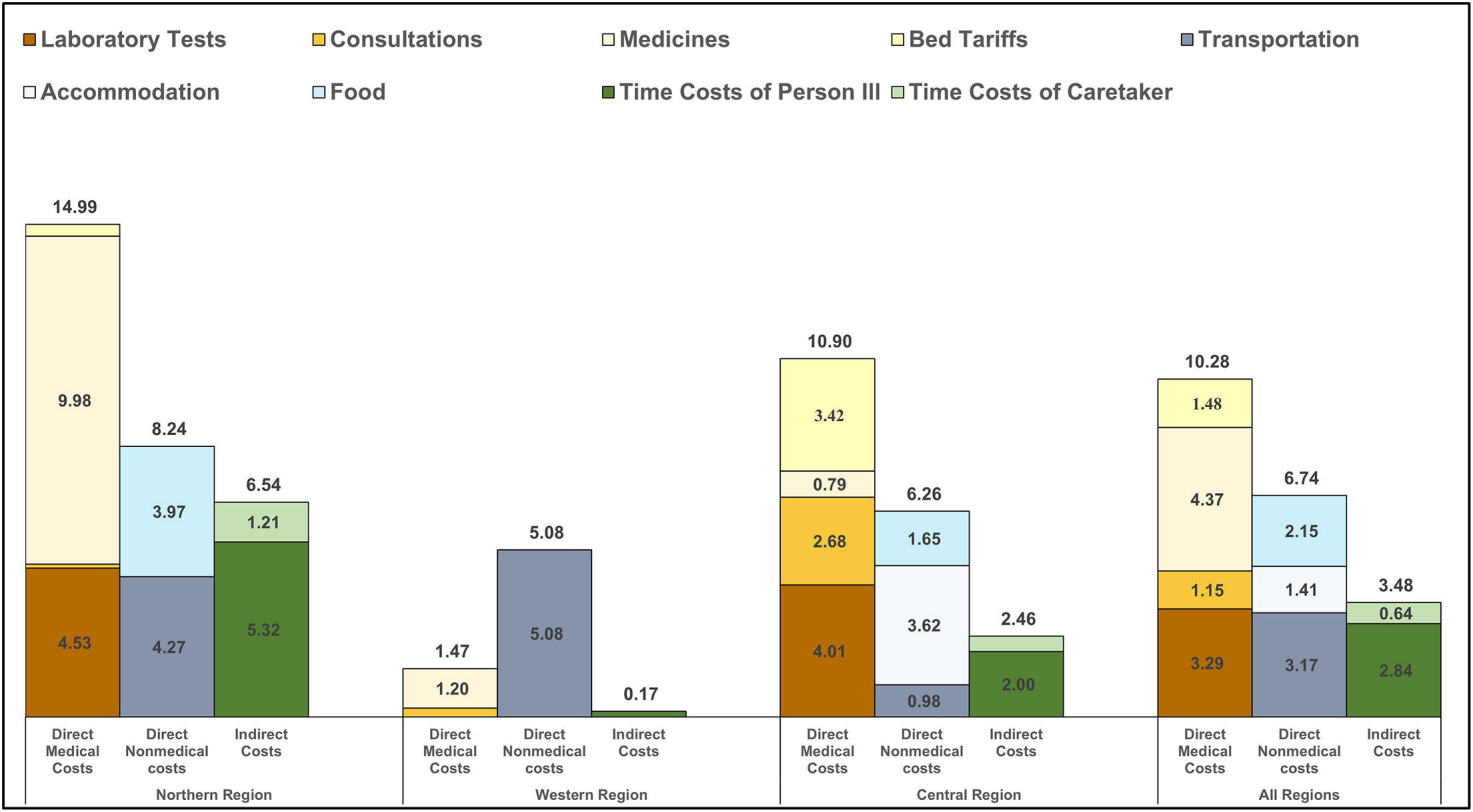
Inpatient costs incurred from receiving care for RHD. The bar graphs show the level and distribution of average costs per person per year for inpatient encounters (*n* = 27). Eleven participants from the Northern Region represented seventeen visits; two participants from the Western Region represented two visits; and eight participants from the Central Region represented eight visits. Costs are disaggregated by region and by component and include direct and indirect costs. Note: costs are presented in 2019 United States dollars.

Direct costs of inpatient care, highlighted in Figure 2, were substantially higher than outpatient costs but were also comprised predominately of medications, laboratory tests and transportation expenses. Indirect costs were a more substantial contributor to inpatient total costs than to outpatient costs. In the setting of limited inpatient cost data, the composition of OOP costs for inpatient care differed across the three regions, with medications, transportation, and laboratory tests costs having outsized importance in the Northern, Western and Central regions, respectively.

### Catastrophic Health Expenditure

Table 3 presents our estimates of the prevalence of catastrophic health expenditure (CHE) in this sample. At the more conservative 25% threshold, 20% of households experienced CHE, ranging from 3% in the Central Region to 32% in the Western Region. At the more liberal 10% threshold, 35% of households experienced CHE, ranging from 9% in the Central Region to 53% in the Western Region. Put another way, households in the Northern and Western regions were five to ten times more likely to experience CHE as compared to households in the Central Region.

**Table 3:** Percentage of households experiencing catastrophic health expenditure by region.

	**Northern region (*n* = 33)**	**Western region (*n* = 19)**	**Central region (*n* = 34)**	** (*N* = 86)**
10% Threshold (%)	52	53	9	35
25% Threshold (%)	30	32	3	20

### Coping Strategies

**Figure 3:**
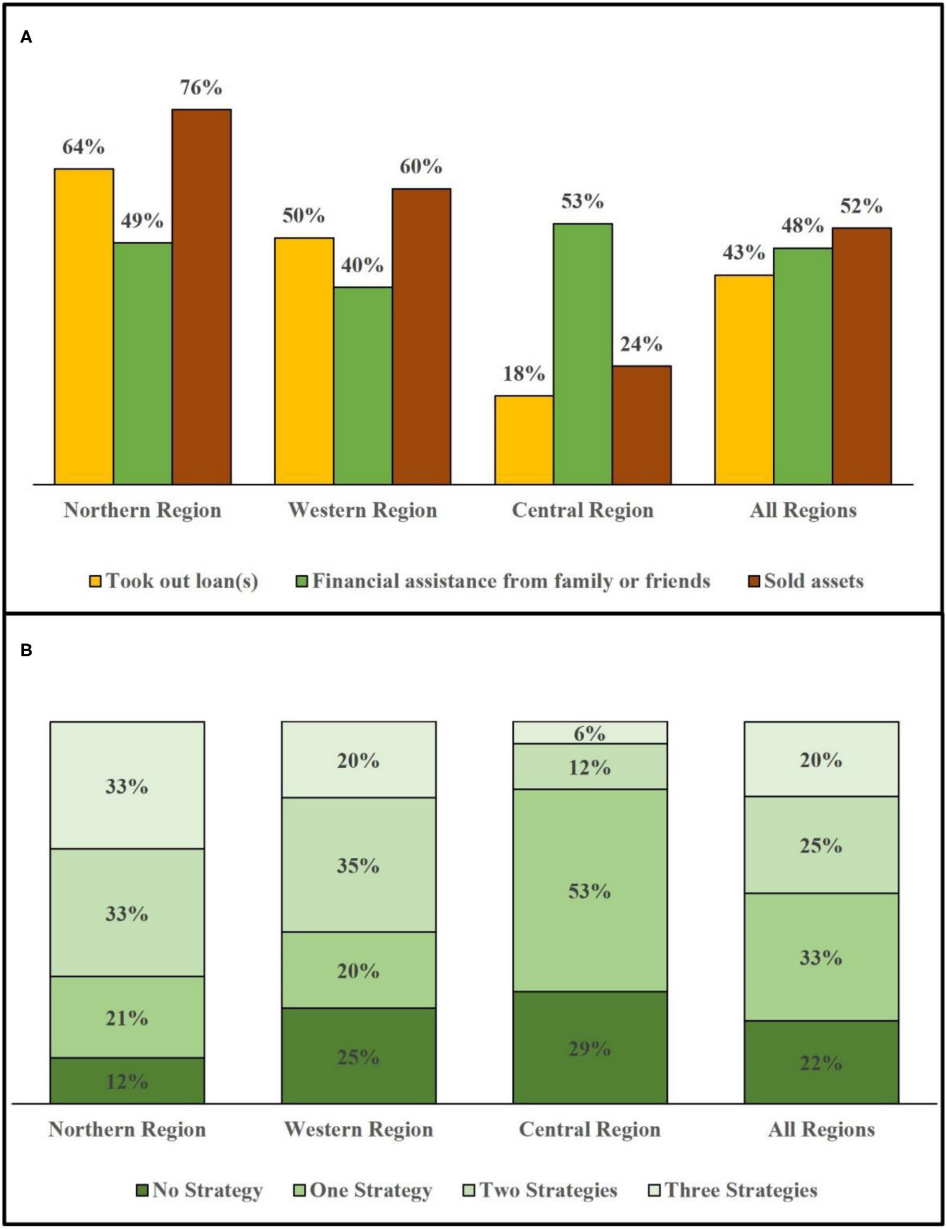
Utilization of financial coping strategies by region. The bar graphs show distribution of financial coping strategies used by households across regions (*n* = 87). This represents the 33 households in the Northern Region, 20 households in the Western Region, and 34 households in the Central Region that were sampled in this study. **(A)** Frequency of each of three coping strategies used. **(B)** Reliance on zero, one, two, or all three coping strategies. Note: Due to rounding, the sum for the Northern region adds to 99%, and the actual number with more decimal points adds to 100%.

Households in the Northern and Western regions demonstrated greater reliance on asset sales and loans than the Central Region, reflecting heavy financial burden due to RHD OOP costs and CHE (Figure 3A). By contrast, participants in the Central Region demonstrated greater reliance on financial assistance from extended family or friends, the latter of which probably reflects greater access to financial resources in the community in this relatively wealthier region.

Across all regions, nearly four out of five households used one or more coping strategies in the past year (Figure 3B). The use of multiple coping strategies was also particularly notable: nearly half of households used at least two coping strategies, and one in five used all three. Again, households in the Northern and Western regions demonstrated a greater reliance on coping strategies, including the use of multiple strategies, compared to the Central Region.

In the regression analysis, we found that the only significant association with coping strategies was the magnitude of the direct OOP costs that patients incurred (Table 4). However, regional differences in utilizing coping strategies that observed in Figure 3B were not statistically significant in regression.

**Table 4:** Effect of demographic and socioeconomic factors on utilization of coping strategies.

	**IRR^a^**	**Robust S.E.^b^**	**95% CI**	***p*-value**
**DEMOGRAPHIC**				
Age	0.9978	0.0044	(0.9892, 1.0064)	0.6105
Gender (reference group: male)	0.8503	0.1063	(0.6655, 1.0863)	0.1945
Secondary education: yes vs. no	0.8053	0.1384	(0.5749, 1.1279)	0.2077
Region (ref: Northern)				
Western	0.8466	0.1452	(0.6049, 1.1848)	0.3315
Central	0.7476	0.1531	(0.5004, 1.1168)	0.1554
**SOCIOECONOMIC**				
Household income per capita	0.9997	0.0004	(0.999, 1.0004)	0.4083
Employment (reference group: unemployed)	0.8796	0.1881	(0.5785, 1.3375)	0.5486
Private insurance: yes vs. no	1.1793	0.3577	(0.6508, 2.1369)	0.5867
Total direct RHD costs	1.0025	0.0005	(1.0014, 1.0035)	0.0000
**INTERCEPT**	1.8023	0.2267	(1.4085, 2.3062)	0.0000

a *Incident rate ratio*,

b *standard error*.

## Discussion

This study sought to quantify the economic cost of RHD-related healthcare among 87 patients and their households from three diverse districts in Uganda. We found that the total annual cost of receiving care for RHD was US$ 78 per patient, inclusive of both direct and indirect costs. Medications and transportation costs appeared to be the major determinants of high OOP costs. About one-third (35%) of households affected by RHD experienced CHE during the past year, and nearly four in five households coped with these costs using some combination of formal and informal borrowing and asset sales. Unsurprisingly, we found a strong association between the magnitude of OOP costs and the probability of using one or more coping strategies. Our research confirms that RHD is a neglected disease of poverty that results in high healthcare costs, distortions in household economic decision-making, and exacerbation of medical poverty traps.

This study is the second manuscript and the first peer-reviewed study to look at the household economic impact of RHD in the African region. Further, this study adds to the global literature documenting the high costs of chronic diseases to households in low- and middle-income countries—costs which are driven by the long-term nature of healthcare and the high costs of medicines, many of which are not publicly financed (22). Previous research by Oyebamiji in South Africa painted a somewhat more optimistic picture that reflects important differences in health system arrangements between South Africa, an upper-middle-income country, and Uganda, a low-income country. For example, none of the subjects in the South African study incurred direct medical costs (due to a robust free healthcare policy), and the prevalence of coping strategies was much lower than in our study (9). These differences show the positive impact that progressive financing policies can have on households affected by RHD and other chronic health conditions.

Consistent with studies of other health conditions in a range of African countries, transportation costs were a major determinant of OOP costs in our study (23, 24). Because RHD prevention and treatment services are only currently offered at regional referral hospitals in Uganda, and often only in the context of research studies, our study subjects often had to travel significant distances and thus incur considerable costs to receive routine services like monthly antibiotic injections. In addition, retention and medication adherence among individuals enrolled in the Uganda National RHD Registry has been reported to be as low as 41%, which means that it is quite possible that distance to health facilities is a major deterrent to high-quality RHD care (25).

Persons affected by RHD are generally young (average age of 28 years in our study) and can experience substantially better lifetime productivity and quality of life when they have access to regular healthcare, including interventional procedures or heart valve surgeries when their condition deteriorates. Our study demonstrates the strong, mutually reinforcing linkage between chronic disease and poverty. For example, three-quarters of the participants in our study were unemployed and usually had to sacrifice many hours per month traveling to and from health facilities. Since RHD is a disease of poverty, cross-cutting efforts to improve healthcare access and affordability among vulnerable populations in Uganda would prove beneficial for persons with RHD.

While this study does not directly impact clinical decision-making surrounding RHD, it has implications for the organization of cardiovascular health services in Uganda and other countries with similar health system arrangements. Specifically, the current model of highly centralized cardiovascular services shifts many of the costs of RHD from the system onto households and therefore requires immediate reform. We conclude that the Uganda Ministry of Health should experiment with decentralized models of care, at least for services that do not require an in-person, specialized workforce, such as the capture of echocardiography images and administration of prophylactic antibiotics (26). This may have implications for clinical training and expected competencies of non-specialist medical providers. For instance, nurses at local health centers could perform routine focused RHD-screening echocardiograms for remote interpretation. This could assist in the triage of secondary prophylaxis and referral to a cardiologist or surgeon at a regional referral health center. Such referrals may at times be accommodated by telehealth to minimize the barrier of geographical distance.

Additionally, the Government of Uganda does not generally charge user fees for services received at public healthcare facilities. But regional hospitals are authorized to run private patient services and therefore are allowed to charge for certain services within the public hospital. Likewise, medications dispensed from these facilities are typically free of charge—in principle. In practice, though, supply chains for medications are weak, and stock-outs are frequent, especially for medications for cardiovascular diseases (27). The absence of essential medications at public facilities pushes patients to seek care at private pharmacies where these medications are more widely available but are offered on a cost-recovery basis. We strongly suspect that this phenomenon explains our findings regarding the contribution of medication costs to the total OOP costs of RHD care. An important first step in ensuring UHC for RHD will be to invest in supply chains for medications related to RHD, many of which are used for other cardiovascular diseases (e.g., beta-blockers and angiotensin-converting enzyme inhibitors) or for infectious diseases (e.g., benzathine penicillin and azithromycin). This investment would represent a modest subset of the total, long-term cost of a comprehensive RHD program in Uganda. It would, however, provide immediate financial protection to individuals already known to have RHD and would pre-emptively address a key bottleneck to program scale-up.

## Limitations

Our study has a number of important limitations. Our sample size was relatively small and was truncated prematurely due to the ongoing global SARS-CoV-2 pandemic. We sampled participants who were receiving care at three regional referral hospitals and who therefore had the ability to travel potentially far distances. While we attempted to capture the range of socioeconomic and health system variations in the country, our estimates are not statistically generalizable to national averages and may not capture those who are unable to travel to receive RHD care. The accuracy of household survey data is known to decline with long recall periods, so we expect some measurement error to exist for events that were reported to have occurred greater than a few months ago (we chose a 12-month recall period to ensure we captured costly but infrequent events such as hospitalizations). Finally, the prevalence-based approach to conducting a cost-of-illness study may miss important dimensions of costs as compared to an incidence-based approach, such as the evolution of costs with disease progression or inter-temporal consumption smoothing. Despite these important limitations, our study provides crucial emerging insights into the economic consequences of RHD in countries where the disease remains endemic. Our approach and data collection tools (Supplementary Material) could prove useful to other researchers, advocates, and health ministries that are seeking to implement the 2018 global resolution on RHD or to build the economic case for greater public investment in RHD control programs.

## Future Directions

Future research on the household economics of RHD (and other chronic diseases) would benefit from the longitudinal approach to further capture how RHD costs and its socioeconomic consequences evolve over time. These studies should further consider the use of cohorts sampled from the general population, rather than disease registries that are biased toward patients with more severe disease and an ability or means to travel long distances.

## Conclusions

We demonstrate that Ugandan households seeking care for RHD incur OOP that are very high relative to income. These costs are enhanced in lower-income regions and exacerbate the poverty trap. To achieve UHC in Uganda, the government will need to enact a series of policy reforms that address the major sources of financial hardship faced by individuals affected by RHD and other chronic diseases.

## Data Availability Statement

The raw data supporting the conclusions of this article is available upon request to the authors, without undue reservation.

## Ethics Statement

This study was approved by the Makerere University School of Medicine Research and Ethics Committee (REC RF 2018-082) and by the Uganda National Council for Science and Technology (SS 5081). Written informed consent to participate in this study was provided by the participants or, in the case of minors, their legal guardian or next of kin. In addition, the University of Washington Human Subjects Division approved an earlier version of this study (STUDY00002855) that did not include subjects under the age of ten.

## Author Contributions

DW conceived the study and acquired funding for the research. YS designed the study with input from DW. JA, RK, and HN collected the data. MN and EN organized the database and supervised the data collection. YD and YK conducted the statistical analysis. AB, EO, DW, and YS reviewed and interpreted the results. CO drafted the first draft of the manuscript. All authors contributed to manuscript revision, read and approved the submitted version.

## Conflict of Interest

The authors declare that the research was conducted in the absence of any commercial or financial relationships that could be construed as a potential conflict of interest.

## Publisher's Note

All claims expressed in this article are solely those of the authors and do not necessarily represent those of their affiliated organizations, or those of the publisher, the editors and the reviewers. Any product that may be evaluated in this article, or claim that may be made by its manufacturer, is not guaranteed or endorsed by the publisher.
