# Supplementary material for: Household Economic Consequences of Rheumatic Heart Disease in Uganda
Source: Front Cardiovasc Med. 2021 Jul 30;8:636280. doi: 10.3389/fcvm.2021.636280 (PMC8363312; doi:10.3389/fcvm.2021.636280)
Supplement: Supplementary file 1 [file Data_Sheet_1.pdf]

Demographics and Contact (Household Survey)

Record ID ______________________________

Interview date and start time ______________________________

Survey Type
○ Baseline
○ 12 Month Follow-Up

SECTION A: PARTICIPANT INFORMATION

A01 District name ______________________________

A01.01 Village ______________________________

A01.02 Parish ______________________________

A01.03 Subcounty ______________________________

A02 Name of index participant ______________________________
(person with RHD enrolled in registry)

A03 Name of household survey respondent (if different than registrant) ______________________________

A04 Contact information

Mobile 1 ______________________________

Mobile 2 ______________________________

Household Roster (Household Survey)

SECTION B: HOUSEHOLD ROSTER (MEMBERS OTHER THAN INDEX PARTICIPANT)
In this section we define a household as a group of people who, over the past 6 months, have lived in the same dwelling space, eaten meals together, shared at least one common plot of land or one activity that generates income, and recognized the authority of one person as the 'head' of the household. Include any adults who fit this definition. Only include children if one of the (adult) household members is their primary caretaker."

"Use the household roster scratch paper to record data, then transfer into the tablet when finished, then save the scratch paper and store it with the paper consent forms."

B01 Line/Roster Number ________________________________

B02 Relationship to head of household
- ◯ Head
- ◯ Spouse
- ◯ Own child
- ◯ Stepchild
- ◯ Grandchild
- ◯ Mother/Father
- ◯ Mother/Father-in-law
- ◯ Grandparent
- ◯ Non-relative
- ◯ Other relative
- ◯ No response

B03 Sex
- ◯ Male
- ◯ Female

B04 Age in completed years ________________________________

B05 Highest grade completed
- ◯ No education
- ◯ Primary
- ◯ Secondary
- ◯ Tertiary - Undergraduate
- ◯ Tertiary - Postgraduate
- ◯ Other
- ◯ Don't know
- ◯ No response

B06 Which of these best describes the household member's work?
- ◯ Full time salary
- ◯ Part time
- ◯ Self-employed
- ◯ Not employed

B07 Does (Name of household member) have health insurance?
- ◯ Yes
- ◯ No
- ◯ Don't know

B08 Does (Name of household member) have any of the following health conditions?

◯ Cancer
◯ Diabetes
◯ HIV/Aids
◯ Tuberculosis
◯ Rheumatic heart disease
◯ Other heart disease
◯ Lung disease
◯ None
◯ No response
(Tick all that apply)

B09 Are there other members of the household? If yes, fill another form

◯ Yes
◯ No

Income and Expenditure (Household Survey)

SECTION C: HOUSEHOLD INCOME.
In this section we shall be asking you about your household monthly wages and if your household has any other sources of income. By wages we mean any payments received for work done over the past 30 days and we are referring to disposable post-tax income.

We would like you to estimate how much income your household received in the last month first, then we will ask about specific sources."

C01 How much in total wages does your household receive per month? ________________________________

"How much money does this household receive form other sources, besides wages, each month? Some examples of non-wage income are inheritance, state grants, and money for child support."

C02.1 - Other Income 1 Description ________________________________

C02.1 Other Income 1 Amount ________________________________

C02.2 - Other Income 2 Description ________________________________

C02.2 Other Income 2 Amount ________________________________

C02.3 - Other Income 3 Description ________________________________

C02.3 Other Income 3 Amount ________________________________

C02.4 Other non-salary income

____

C03 Who is the primary income earner in the household? (enter roster number from section B) ________________________________

C04 How much does the primary income earner earn per month? ________________________________

C05 Does anyone else in the house earn income ?
- ◯ Yes
- ◯ No
- ◯ Don't Know

If yes how much do they earn each month, on average?

Please list up to 7 members and include their roster number and monthly income amount.

C05.01.01 Member 1 Roster Number ________________________

C05.01.02 Member 1 Monthly Income ________________________

C05.02 Member 2 Roster Number ________________________

C05.02.01 Member 2 Monthly Income ________________________

C05.03 Member 3 Roster Number ________________________

C05.03.01 Member 3 Monthly Income ________________________

C05.04 Member 4 Roster Number ________________________

C05.04.01 Member 4 Monthly Income ________________________

C05.05 Member 5 Roster Number ________________________

C05.05.01 Member 5 Monthly Income ________________________

C05.06 Member 6 Roster Number ________________________

C05.06.01 Member 6 Monthly Income ________________________

C05.07 Member 7 Roster Number ________________________

C05.07.01 Member 7 Monthly Income ________________________

C06 Was your household income substantially higher before (NAME OF INDEX PARTICIPANT) was diagnosed with RHD?
- ◯ Yes
- ◯ No
- ◯ Don't know

C06.01 If so, about how much more income did your household have per month? ________________________

SECTION D: HOUSEHOLD ASSETS

D01 How does your household dispose of sewage and refuse?
- ◯ Waste vehicle collectors
- ◯ Public incinerator
- ◯ Public grounds
- ◯ Other

D01.01 If other specify ________________________

D02 How is the drainage system in your household?
- ◯ Running
- ◯ Stagnant

D03 What kind of toilet facility do members of your household use?

- ◯ Flush to piped sewer system
- ◯ Flush to septic tank
- ◯ Flush to pit latrine
- ◯ Flush to somewhere else
- ◯ don't know where
- ◯ Ventilated improved pit latrine
- ◯ Pit latrine with slab
- ◯ Pit latrine without slab/open pit
- ◯ Composting toilet
- ◯ Bucket toilet
- ◯ Hanging toilet/hanging latrine
- ◯ No facility, bush, field
- ◯ Other

D04 Do you share this toilet facility with other households?

- ◯ Yes
- ◯ No

D05 Does your household have?

	Yes	No
D05.01 Electricity	☐	☐
D05.02 Radio	☐	☐
D05.03 Television set	☐	☐
D05.04 Mobile phone	☐	☐
D05.05 Non-mobile phone	☐	☐
D05.06 Refrigerator	☐	☐
D05.07Cable or Satellite TV	☐	☐
D05.08 Generator	☐	☐
D05.09 Computer	☐	☐
D05.10 Electric iron	☐	☐
D05.11 Fan	☐	☐

D06 What material is used to construct the floor of your house?

- ◯ Earth/sand
- ◯ Dung
- ◯ Wood planks
- ◯ Parquet or polished wood
- ◯ Vinyl or asphalt strips
- ◯ Ceramic tiles
- ◯ Cement
- ◯ Carpet
- ◯ Other
- ◯ Don't know
- ◯ Refuses to respond

D07 What material is used to construct the roof of your house?

- ◯ No roof
- ◯ Thatch/palm leaf
- ◯ Sod
- ◯ Rustic mat
- ◯ Palm/bamboo
- ◯ Wood planks
- ◯ Cardboard
- ◯ Corrugated iron sheets
- ◯ Metal
- ◯ Wood
- ◯ Calamine/cement fiber
- ◯ Other
- ◯ Don't know
- ◯ Refuses to respond

D08 What material is used to construct the exterior walls of your house?

- ◯ No walls
- ◯ Cane/palm/trunks
- ◯ Dirt
- ◯ Rudimentary walls
- ◯ Bamboo with mud
- ◯ Stone with mud
- ◯ Uncovered adobe
- ◯ Plywood
- ◯ Cardboard
- ◯ Reused walls
- ◯ Cement
- ◯ Stone with limit/cement
- ◯ Bricks
- ◯ Cement blocks
- ◯ Uncovered adobe
- ◯ Wood planks/shingles
- ◯ Other
- ◯ Don't know
- ◯ Refuses to respond

D09 How many rooms are used for sleeping in your household?

____

D10 How many people sleep together in a room in your household on average?

- ◯ One person
- ◯ Two persons
- ◯ Three persons
- ◯ Four persons
- ◯ Five persons
- ◯ More than five persons
- ◯ Don't know/ refuses to respond

D11 Does any member of this household own?

	Yes	No
D11.01 A watch	◯	◯
D11.02 A bicycle	◯	◯
D11.03 A motorcycle	◯	◯
D11.04 Animal drawn cart	◯	◯
D11.05 A car	◯	◯

D11.06 A boat with a motor	◯	◯
D11.07 A canoe	◯	◯
D11.08 Agricultural land	◯	◯

D12 If you answered yes to agricultural land, how many acres/plots are owned? ______________________

D12.01 Please specify metric (acre, plot, etc.) ______________________

D13 Does this household own any livestock, herds, other farm animals, or poultry?
◯ Yes
◯ No

D14 How many of the following animals does your household own? Enter "0" if none.

D14.01 Milk cows or bulls ______________________

D14.02 Goats ______________________

D14.03 Sheep ______________________

D14.04 Chickens/Ducks ______________________

D14.04 Pigs ______________________

D14.05 Other livestock

____

SECTION E: HOUSEHOLD EXPENDITURES

E01 In a typical week, how much does your household usually spend on food? ______________________

E02 Within the last 30 days, did you receive any foodstuffs as payment?
◯ Yes
◯ No
◯ Don't know

E03 Within the last 30 days, did you produce/grow/gather any foodstuffs?
◯ Yes
◯ No
◯ Don't know
◯ Refuse to respond

E04 Within the last 30 days, did you eat food from your own stock shop or bought at the mall?

	Yes	No	Don't know
E04.01 Own stock shop	◯	◯	◯
E04.02 Stock shop	◯	◯	◯
E04.03 Mall	◯	◯	◯

E05 Over the last 30 days, how much in total did your household spend on goods and services apart from food? ______________________

E06 Within the last 30 days, about how much did your household spend on the following?

E06.01 Transportation ______________________

E06.02 Utilities ______________________

E06.03 Household Items ______________________

How much was spent over the past 30 days on the following transportation categories?

E06.01.01 Car payments excluding insurance ______________________

E06.01.02 Petrol, oil and car service ______________________

E06.01.03 Buses, taxis boda boda, trains and air tickets including transport to school, work etc ______________________

How much was spent over the past 30 days on the following utility categories?

E06.02.01 Water ______________________

E06.02.02 Electricity ______________________

E06.02.03 Other energy sources such as wood, paraffin, charcoal/coal, candles, gas, batteries, or diesel oil for generators ______________________

How much was spent over the past 30 days on the following household items categories?

E06.04.01 Kitchen equipment, like pots and pans, cutlery and crockery ______________________

E06.04.02 Home maintenance and repairs to the dwelling ______________________

E06.04.03 Bedding, sheets, blankets and towels ______________________

How much was spent over the past 30 days on items in following the miscellaneous categories?

E06.05.01 Washing powder, dishwashing liquid, polish and all household cleaners ______________________

E06.05.02 Religious and membership dues of organisations, donations to charity ________________________________

E06.05.03 Other 1 Description ________________________________

E06.05.03 Other 1 Amount ________________________________

E06.05.04 Other 2 Description ________________________________

E06.05.04 Other 2 Amount ________________________________

E06.05.05 Other 3 Description ________________________________

E06.05.05 Other 3 Amount ________________________________

SECTION F: INTRA-HOUSEHOLD EFFECTS OF RHD

F01 Who is the primary caregiver of the Participant? (household roster code number) ________________________________

F02 Has this primary caregiver given up any of the following to take care of the participant?

F02.01 Number of days of school missed in the last 12 months ________________________________

F02.02 Number of hours of work given up per week ________________________________

F02.03 Other given up (describe) ________________________________

F02.04 Other given up (hours per week) ________________________________

F03 Has this person had to significantly reduce the number of hours above to take care of index participant?
- ◯ Yes
- ◯ No
- ◯ Don't know

F03.01 If yes to the question above, approximately how many hours per week have been lost? For example if you used to work 40 hours per week but now work 30 hours per week answer "10." ________________________________

F04 Other household members

F04.01 Has anyone given up school to take care of the patient?
- ◯ Yes
- ◯ No
- ◯ Don't know

F04.01.01 If yes Who? ________________________________
(household roster numbers)

F04.01.02 How many days of school were missed in total over the past 12 months? ________________________________
(sum of days overall response)

F4.02 Has anyone given up work to take care of the patient?

◯ Yes
◯ No
◯ Don't know

F04.02.01 If yes who?

____
(household roster numbers)

F04.02.02 On average how many hours per week have been given up?

____
(sum of hours over all responses)

Form completion status (Household survey)

Form status

Interview stop time ________________________________

Demographics and Contact (Individual Survey)

Record ID ______________________

Date and time of interview ______________________

SECTION A: DEMOGRAPHICS

A01 Sex
- ◯ Male
- ◯ Female

A02 Age in completed years ______________________

A03 Marital status
- ◯ Single/never married
- ◯ Married
- ◯ Widow/widower
- ◯ Separated/divorced
- ◯ Prefer not to respond

A04 Education
- ◯ No education
- ◯ Primary
- ◯ Secondary
- ◯ Tertiary - Undergraduate
- ◯ Tertiary - Postgraduate
- ◯ Other
- ◯ Don't know
- ◯ No response

(highest grade completed)

A05 Employment
- ◯ Full time salary
- ◯ Part time
- ◯ Self-employed
- ◯ Not employed

A06 Relationship to head of household
- ◯ Head
- ◯ Spouse
- ◯ Own child
- ◯ Step child
- ◯ Grandchild
- ◯ Mother/Father
- ◯ Mother/Father-in-law
- ◯ Grandparent
- ◯ Non-relative
- ◯ Other realative

A07 Do you have any of the following co-morbid conditions?
- ◯ Cancer
- ◯ Diabetes
- ◯ HIV/AIDS
- ◯ Tuberculosis
- ◯ Other heart disease
- ◯ Lung disease
- ◯ None

(Tick all that apply)

Disability (Individual Survey)

SECTION B: DISABILITY

Thinking about your overall level of health, which of these statements best describes

B01 Mobility	○ no problems in walking about	○ some problems in walking about	○ confined to bed
B02 Self care	○ no problems with self-care	○ some problems washing or dressing myself	○ unable to wash or dress myself
B03 Usual Activities (e.g. work, study, housework, family or leisure activities)	○ no problems with performing my usual activities	○ some problems with performing my usual activities	○ unable to perform my usual activities
B04 Pain or discomfort	○ no pain or discomfort	○ moderate pain or discomfort	○ extreme pain or discomfort
B05 Anxiety/Depression	○ not anxious or depressed	○ moderately anxious or depressed	○ extremely anxious or depressed

Income and Insurance (Individual Survey)

SECTION C: INDIVIDUAL INCOME
In this section we shall be asking about your monthly wages, any other sources of income and insurance. By wages we mean any payments received for work done over the past month. We are referring to disposable income (post-tax).

C01 How much in total wages do you receive per month? __________________________________
(monthly income; if unemployed enter 0)

"How much money do you receive from other sources, besides wages, each month? Some examples of non-wage income are inheritance, state grants, and money for child support."

C02.01 - Other Income 1 Description __________________________________

C02.01 Other Income 1 Amount __________________________________

C02.02 - Other Income 2 Description __________________________________

C02.02 Other Income 2 Amount __________________________________

C02.03 - Other Income 3 Description __________________________________

C02.03 Other Income 3 Amount __________________________________

C02.04 Other non-salary income ____

C03 Health insurance

C03.01 Do you have private health insurance?
- ◯ Yes
- ◯ No
- ◯ Don't know

C03.02 Is your insurance suffient for RHD care?
- ◯ Yes
- ◯ No
- ◯ Don't know
- ◯ Not applicable

C03.03 Do you face any challenges related to your health insurance? If so please explain briefly.
____

Cost of Outpatient Visits (Individual Survey)

Have you been to the clinic for RHD in the past 12 months?

◯ Yes
◯ No
◯ Don't know

If yes continue with section D

SECTION D: COST OF OUT PATIENT CARE

"In this section we shall be asking about the money and time spent on your RHD clinic visits over the past 12 months. We will ask separately about any inpatient admissions for RHD over the past 12 months."If you have any receipts or invoices related to your clinic expenses,we would like to see those to document the correct amount,if at all possible. If receipts are not available please provide your best estimate"
We would like to start with today's visit and work backwards"

D01 Thinking back to your most recent clinic visit for Rheumatic Heart Disease (starting with today) What day/month did you visit? ________________________________

D02

"We want to know how much time and money you expect you will have spent between the time you left your home to come to the clinic and the time you return home later, excluding the time taken for this interview. So for instance, if you home at 8am this morning, and it is now 3.30pm and it will take you an hour to get back home, we want to know how you will have used your time between 8am and 5pm today and how much money was spent during that time."

"Bearing that in mind, how much did you spend on:"

D02.01 Laboratory (blood draw e.t.c) ________________________________

D02.02 Consultation ________________________________

D02.03 Medications ________________________________

D02.04 Transport ________________________________

D02.05 Accomodation ________________________________

D02.06 Food and Other Expenses ________________________________

D03

How much time was spent during the last visit on:

D03.01 Transport to facility ______________________

D03.02 Waiting time ______________________

D03.03 Time with provider ______________________

D03.04 Estimated transportation back ______________________

D04 How much time did you miss from work? ______________________

D05 how much time did your child miss from school? ______________________

D06 Did anyone accompany you during your last visit?
- ◯ Yes
- ◯ No

If yes who and how many?
For example if 1 parent and 2 siblings accompanied the participant write "1" for parent and "2" for siblings.

Write "0" for the responses below if none of them accompanied the participant.

D06.01 Parent/ guardian ______________________

D06.02 Sibling ______________________

D06.03 Child ______________________

D06.04 Neighbor/ friend/ other caretaker ______________________

D06.05 Other ______________________

D07 In total how many days of work or school did this/these person/persons have to miss in order to accompany you?
"For example if 2 siblings accompanied the participant and each missed 2 days of school, then the total number of days of school missed is 4."

D07.01 Total number of days of work missed ______________________
(Sum of all individuals missing work)

D07.02 Total number of days of school missed ______________________
(Sum of all individuals missing school)

D08 Have you had any other RHD health center visits over the past 12 months?
- ◯ Yes
- ◯ No
- ◯ Don't know

If yes, complete another form.

Cost Of Inpatient Visits (Individual Survey)

SECTION E: COST OF INPATIENT CARE
" In this section we will be asking about the money and time spent on your RHD inpatient visits over the past 12 months. We would like to start with today's visit (the most recent visit) and work backwards."

"If you have any receipts or invoices related to your hospital expenses we would like to see those, if at all possible to document the correct amount. If receipts are not available please provide your best estimate."

E01 To your knowledge, have you been hospitalized for RHD in the last 12 months?
◯ Yes
◯ No
◯ Don't know

E01.01 If yes to the above what month/year were you hospitalized? ______________________________

E01.02 How many days were you hospitalized? ______________________________

E01.03 Did you have heart surgery while you were in hospital?
◯ Yes
◯ No
◯ Don't know

E02 For the visit described above, how much was spent on?

E02.01 Laboratory (blood, X-ray etc) ______________________________

E02.02 Consultation fees ______________________________

E02.03 Medications ______________________________

E02.04 bed tariffs or other hospital charges ______________________________

E02.05 Transport ______________________________

E02.06 Accomodation ______________________________

E02.07 Food and Other Expenses ______________________________

E03.01 How many days of work did you have to miss? ______________________________

E03.02 How many days of school did you have to miss? ______________________________

F04 Other Household Members

F04.01 Has anyone given up school to care for the patient?
- ◯ Yes
- ◯ No
- ◯ Don't know

E04.02 Person 1 Missed School Roster Number ______________________

E04.03 Person 2 Missed School Roster Number ______________________

E04.04 Person 3 Missed School Roster Number ______________________

E04.05 Person 4 Missed School Roster Number ______________________

E04.06 How many days of school were missed in total over the past 12 months? (sum of days over all responses) ______________________

F05.01 Has anyone given up work to care for the patient?
- ◯ Yes
- ◯ No
- ◯ Don't know

E05.02 Person 1 Missed Work Roster Number ______________________

E05.03 Person 2 Missed Work Roster Number ______________________

E05.04 Person 3 Missed Work Roster Number ______________________

E05.05 Person 4 Missed Work Roster Number ______________________

E05.06 On average, how many hours per week have been given up? (sum of over all responses) ______________________

F06.01 Has anyone taken on extra work to pay RHD expenses?
- ◯ Yes
- ◯ No
- ◯ Don't know

E06.02 Person 1 Extra Work Roster Number ______________________

E07 Were you hospitalized for RHD any other time in the past 12 months?
- ◯ Yes
- ◯ No

If yes fill another form

Coping strategies (Individual Survey)

SECTION F: COPING STRATEGIES

F01.01 In the past year, have you ever had to take out a loan to pay for medical expenses related to RHD?
- ◯ Yes
- ◯ No
- ◯ Don't know

F01.02 If yes how much was the loan worth? ______________________

F01.03 Does the loan have interest?
- ◯ Yes
- ◯ No
- ◯ Don't know

F01.04 What is the interest rate on the loan (%)? ______________________

F02 Did you receive financial assistance from friends and family?
- ◯ Yes
- ◯ No
- ◯ Don't know

F02.01 Was this financial assistance a loan or gift?
- ◯ Loan
- ◯ Gift

F02.02 How much was the loan or gift worth? ______________________

F03 Has this illness caused you to reduce the number of hours spent at school?
- ◯ Yes
- ◯ No
- ◯ Not in school

F03.01 Were you ever forced to be expelled from school on basis of your illness?
- ◯ Yes
- ◯ No
- ◯ Don't Know
- ◯ Wish Not to Answer

F03.02 Briefly explain and attempt to quantify how much your illness has set you back in school (for example, held back one grade, had to repeat one subject, missed three weeks last year, etc) ______________________

F04 Has this illness in the past 12 months caused you to reduce the number of hours spent at work?
- ◯ Yes
- ◯ No
- ◯ Not working

F04.01 If yes, how many hours per week were you working before and after the reduction? ______________________

F04.02 Have you ever needed to resign or have you ever been fired on basis of your illness?
- ◯ Yes
- ◯ No

F05 Have you sold any of your property to pay for RHD care?
- ◯ Yes
- ◯ No
- ◯ Don't know

F05.01 If yes, what did you sell?
- ☐ Land
- ☐ Livestock
- ☐ Transport/vehicle
- ☐ Household item
- ☐ Farm produce
- ☐ Other

F05.01.01 If other specify __________________________________

F05.01.02 How much did you earn from the sale? __________________________________

F05.01.03 How much in total would you estimate the property is worth? (If different from the amount of money you sold it for) __________________________________

Form completion status (Individual survey)

Form status

Interview stop time ________________________________
